# More new deep-reef basslets (Teleostei, Grammatidae, *Lipogramma*), with updates on the eco-evolutionary relationships within the genus

**DOI:** 10.3897/zookeys.729.21842

**Published:** 2018-01-16

**Authors:** Carole C. Baldwin, Luke Tornabene, D. Ross Robertson, Ai Nonaka, R. Grant Gilmore

**Affiliations:** 1 Department of Vertebrate Zoology, National Museum of Natural History, Smithsonian Institution, Washington, DC, 20560, USA; 2 School of Aquatic and Fishery Sciences, Burke Museum of Natural History and Culture, University of Washington, Seattle, WA, USA; 3 Smithsonian Tropical Research Institute, Balboa, Republic of Panama; 4 Estuarine, Coastal and Ocean Science, Inc., 5920 First Street SW, Vero Beach, FL, 32968, USA

**Keywords:** Caribbean Sea, manned submersible, cryptic species, integrative taxonomy, phylogeny, ocean exploration, Deep Reef Observation Project (DROP)

## Abstract

Two new *Lipogramma* basslets are described, *L. barrettorum* and *L. schrieri*, captured during submersible diving to 300 m depth off Curaçao, southern Caribbean. Superficially resembling *L. robinsi* in having 11–12 bars of pigment on the trunk, *L. barrettorum* is distinct from *L. robinsi* in having a stripe of blue-white pigment along the dorsal midline of the head (vs. a cap of yellow pigment), in patterns of pigment on the median fins, and in having 8–10 gill rakers on the lower limb of the first arch (vs. 11–12). *Lipogramma schrieri* is distinct from all congeners in having seven or eight dark bars of pigment on the trunk and broad, irregular, whitish blue markings on the dorsal portion of the head. The new species are genetically distinct from one another and from seven other *Lipogramma* species for which genetic data are available. A phylogenetic hypothesis derived from mitochondrial and nuclear genes suggests that the new species belong to a clade that also comprises *L. evides* and *L. haberi.* Collectively those four species are the deepest-living members of the genus, occurring at depths predominantly below 140 m. This study thus provides further evidence of eco-evolutionary correlations between depth and phylogeny in Caribbean reef fishes. Tropical deep reefs are globally underexplored ecosystems, and further investigation of Caribbean deep reefs undoubtedly will provide samples of species for which no genetic material currently exists and reveal more cryptic species diversity in the genus.

## Introduction

Baldwin et al. (2016) described two new species of western Atlantic *Lipogramma* basslets collected during diving to 300 m by the *Curasub* submersible, as part of the ongoing Deep Reef Observation Project (DROP) in the southern Caribbean. That brought the total number of species in the genus to 10. However, they noted that their deep-reef collections included two additional putative new species that superficially resemble *L. robinsi* Gilmore 1997. Further investigation has confirmed that these two species represent additional cryptic diversity in the Grammatidae, a family of usually small, brightly colored fishes restricted to deep reefs of the tropical northwest Atlantic Ocean. Here we describe the two new species of *Lipogramma* based on integrated morphological and molecular data, provide a revised phylogenetic hypothesis of relationships within the genus that includes *L. regia* (which we recently caught using the *Curasub* off St. Eustatius), comment on the eco-evolutionary history of the group based on the phylogenetic pattern of species’ depth distributions, and present a revised key to the species of the genus.

## Methods

**Collecting and morphology.** Basslets were collected using Substation Curaçao’s manned submersible *Curasub* (http://www.substation-curacao.com). The sub has two flexible, hydraulic arms, one of which is equipped with a quinaldine/ethanol-ejection system and the other with a suction hose. Anesthetized fish specimens were captured with the suction hose, which empties into a vented plexiglass cylinder attached to the outside of the sub. At the surface, the specimens were photographed, tissue sampled, and fixed in 10% formalin. Measurements were made weeks to months after fixation and subsequent preservation in 75% ethanol and were taken to the nearest 0.1 mm with dial calipers or an ocular micrometer fitted into a Wild stereomicroscope. Selected preserved specimens were later photographed to document preserved pigment pattern and X-rayed with a digital radiography system. Images of parasitic cysts were made using a Zeiss Axiocam on a Zeiss Discovery V12 SteREO microscope. Counts and measurements follow Hubbs and Lagler (1947). Symbolism for configuration of supraneural bones, anterior neural spines, and anterior dorsal pterygiophores follows Ahlstrom et al. (1976): **USNM** = Smithsonian Institution, National Museum of Natural History; **UF** = Florida Museum of Natural History.

**Molecular analyses.** Tissue samples for 98 specimens assignable to nine species of *Lipogramma* were used for molecular analyses (Appendix 1). Tissues of *L. robinsi, L. rosea* Gilbert, 1979 (in Robins and Colin 1979), and *L. flavescens* Gilmore & Jones, 1988 were not available. Tissues were stored in saturated salt-DMSO (dimethyl sulfoxide) buffer (Seutin et al. 1991). DNA extraction and cytochrome *c* oxidase subunit I (COI) DNA barcoding were performed for 98 specimens (i.e., for all available specimens except one *L. anabantoides* – Appendix 1) as outlined by Weigt et al. (2012). Four nuclear markers were amplified and sequenced—TMO-4C4, Rag1, Rhodopsin, and Histone H3—for 19 specimens of *Lipogramma*, and one or more of those genes was sequenced for an additional three specimens (Appendix 1). Primers and PCR conditions for the nuclear markers followed Lin and Hastings (2011, 2013). Sequences were assembled and aligned using *Geneious v. 9* (Biomatters, Ltd., Aukland). A neighbor-joining (NJ) network was generated for the COI data using the K2P substitution model (Kimura 1980) in the tree-builder application in *Geneious*. Mean within- and between-species K2P genetic distances were calculated from the COI data in *MEGA v. 7* (Kumar et al. 2015). Genetic distances were considered as corroborating morphology-based species delineation if the distances between species were ten or more times the intraspecific differences (Hebert et al. 2004). The alignments of COI and nuclear genes were concatenated and phylogeny was inferred using Bayesian Inference (BI), partitioning by gene. For the Bayesian analysis, substitution models and partitioning scheme were chosen using PartitionFinder (Lanfear et al. 2012) according to Bayesian Information Criterion scores. The chosen scheme had the following partitions and models: COI, HKY+I+G; Histone H3 plus Rhodospin, HKY+G; TMO-4C4, K80+G; Rag1, K80+G. All partitions in the ML analysis received a GTR-GAMMA substitution model. The BI phylogeny was inferred in the program *MrBayes v. 3.2* (Ronquist et al. 2012) using two Metropolis-coupled Markov Chain Monte Carlo (MCMC) runs, each with four chains. The analysis ran for 10 million generations sampling trees and parameters every 1000 generations. Burn-in, convergence and mixing were assessed using Tracer (Rambaut and Drummond 2007) and by visually inspecting consensus trees from both runs. Outgroups for the phylogenetic analysis included two species of *Gramma* and several other genera from the Ovalentaria *sensu* Wainwright et al. (2012): *Acanthemblemaria* (Chaenopsidae), *Helcogramma* (Tripterygiidae), *Blenniella* (Blenniidae), and *Tomicodon* (Gobiesocidae).

To further corroborate the morphologically diagnosed species using our molecular data, we conducted a coalescent-based, Bayesian species-delimitation analysis (Yang and Rannala 2010, 2014). We used the computer program BP&P ver. 3.2 (Bayesian Phylogenetics and Phylogeography – Yang and Rannala [2010], Yang [2015]), which analyzes multi-locus DNA sequence alignments under the multispecies coalescent model (Rannala and Yang 2003). We used the five DNA alignments for the 22 *Lipogramma* specimens in BP&P, with each sequence in the alignments being assigned to one of nine groups *a priori*, based on diagnostic features of morphology and pigmentation. BP&P was then used to simultaneously infer a species tree and calculate posterior probabilities of different species-delimitation models, i.e., models comprising nine species, fewer than nine species (lumping multiple “morpho-species”), or more than nine species (splitting “morpho-species”).

**Depth distributions.** We updated the depth histogram for *Lipogramma* of Baldwin et al. (2017: fig. 10) with the new-species names (originally listed as “*L. ‘robinsi*’ sp. 1” and “*L. ‘robinsi*’ sp. 2”) and with new depth information for the new species and for *L. regia* based on submersible-caught specimens. Additionally, with resolution of the “*L. robinsi*” complex, we added *L. robinsi* based on depth information in the original description (Gilmore 1977).

**Accession numbers.** GenSeq nomenclature (Chakrabarty et al. 2013) and GenBank accession numbers for DNA sequences derived in this study are presented along with museum catalog numbers for voucher specimens in Appendix 1.

## Taxonomy

### Lipogramma barrettorum

Taxon classificationAnimaliaORDOFAMILIA

Baldwin, Nonaka & Robertson sp. n.

http://zoobank.org/B73F04C1-DBEB-4172-8E6E-71AC9625E76C

**Figure 1.**
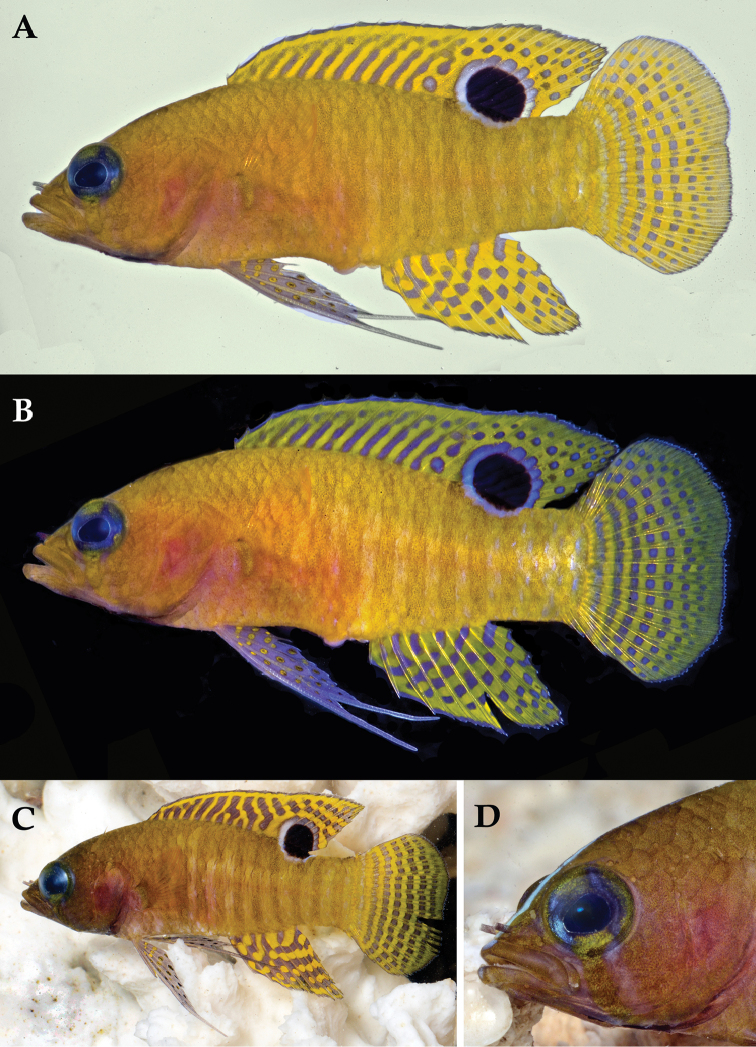
*Lipogramma barrettorum* sp. n. UF 239254, CUR11426, paratype, 28.0 mm SL **A** photographed against a light background and **B** against a dark background. Photographs by D. R. Robertson and C. C. Baldwin. **C** and **D** USNM 440439, CUR16008, holotype, 26.5 mm SL, aquarium photographs by Barry Brown.

**Figure 2.**
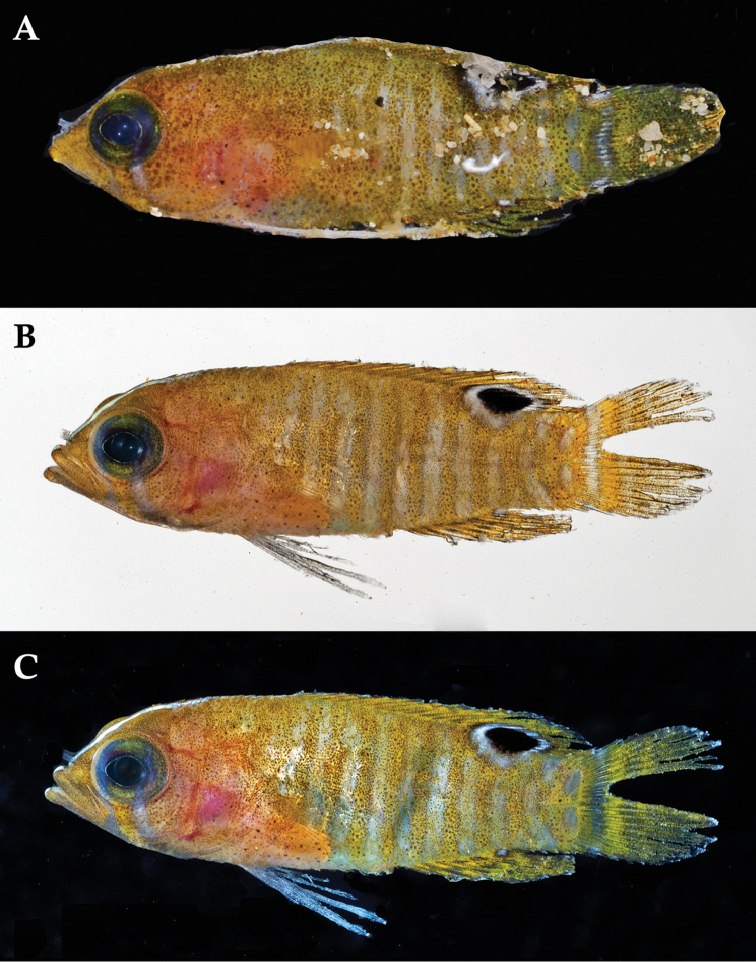
*Lipogramma barrettorum* sp. n. **A** USNM 436474, CUR15139, paratype, 10.2 mm SL **B** and **C** USNM 414914, CUR12149, paratype, 13.0 mm SL, photographed against light (**B**) and dark (**C**) backgrounds. Photographs by D. R. Robertson and C. C. Baldwin.

**Figure 3.**
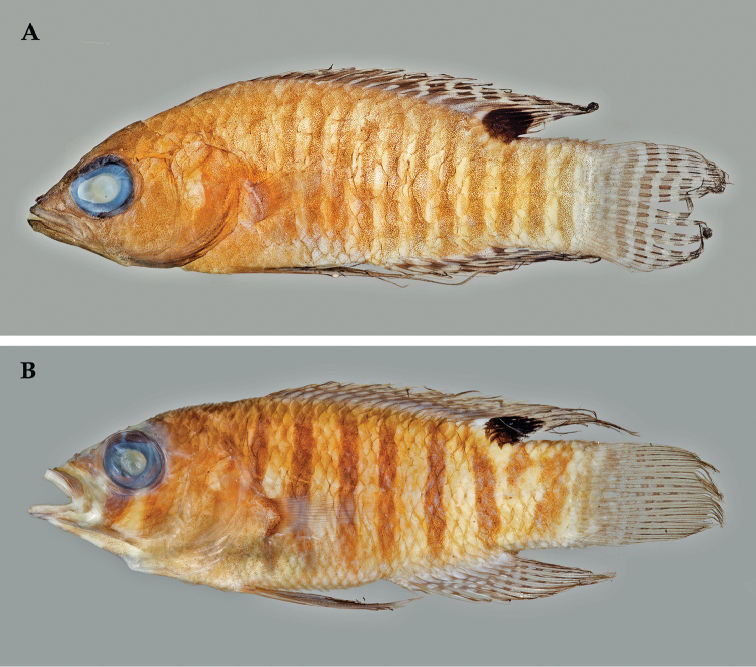
Preserved holotypes of new *Lipogramma* species **A** *L. barrettorum*, USNM 440439, CUR16008, 26.5 mm SL **B** *L. schrieri*, USNM 431722, CUR14114, 49.7 mm SL. Photographs by C. C. Baldwin.

English: Blue-Spotted Basslet; Spanish: Cabrilleta manchado azul Figures 1 , 2 , 3

#### Type locality.

Curaçao, southern Caribbean.

#### Holotype.

USNM 440439, 26.5 mm SL, tissue no. CUR16008, GenBank accession no. MG676227, Curasub submersible, sta. CURASUB16-33, Curaçao, west of Substation Curaçao downline, 12.083197 N, 68.899058 W, 161 m depth, 7 October 2016, C. Baldwin, B. Van Bebber, D. Pitassy & T. Devine.

#### Paratypes.

USNM 406392, 24.5 mm SL, tissue no. CUR11392, Curasub submersible, sta. CURASUB11-06, Curaçao, off Substation Curaçao, 12.083197 N, 68.899058 W, 132–141 m depth, 31 May 2011, C. Baldwin, B. Van Bebber, A. Schrier & A. Driskell; UF 239254, 28.0 mm SL, tissue no. CUR11426, collection information same as USNM 406392; USNM 414914, 13.0 mm SL, tissue no. CUR12149, Curasub submersible, sta. CURASUB12-15, Curaçao, off Substation Curaçao, 12.083197 N, 68.899058 W, 123–160 m depth, 10 August 2012, C. Baldwin, B. Brandt, A. Schrier & P. Mace; USNM 431687, 25.2 mm SL, tissue no. CUR14079, Curasub submersible, sta. CURASUB-MISC14, Curaçao, off Substation Curaçao, 12.083197 N, 68.899058 W, no depth data, September 2014, Substation Curaçao staff; USNM 436460, 27.0 mm SL, Tissue no. CUR15125, Curasub submersible, sta. CURASUB15-21, Curaçao, off Substation Curaçao, 12.083197 N, 68.899058 W, 90–249 m depth (no discrete depth observation), 22 September 2015, C. Baldwin, B. Brandt & E. Duffy; USNM 436474, 10.2 mm SL, tissue no. CUR15139, Curasub submersible, sta. CURASUB15-27, Curaçao, Playa Forti, 12.368 N, 69.155 W, 50–246 m (no discrete depth observation), 29 September 2015, A. Collins, B. Brandt, A. Schrier & T. Devine.

#### Diagnosis.

A species of *Lipogramma* distinguishable from congeners by the following combination of characters: pectoral-fin rays 15–16 (modally 16); gill rakers 12–14 (modally 12, 8–10 rakers on lower limb); four supraorbital pores present along dorsal margin of orbit, a pore present between one above mid orbit and one above posterodorsal corner of orbit; caudal fin rounded; body mostly yellow in life with 11 or 12 narrow brownish bars on trunk; posterior base of soft dorsal fin with large white- or blue-rimmed black ocellus; dorsal, anal and caudal fins yellow with blue/grey (brown in preservative) wavy bars or square-shaped spots. Pelvic fins blue/grey with scattered yellow-ringed dark spots. The new species is further differentiated genetically from congeners for which molecular data are available in mitochondrial COI and nuclear Histone 3, Rhodopsin, TMO-4C4, and RAG1.

#### Description.

Counts and measurements of type specimens given in Table 1. Seven specimens examined, 10.2–28.0 mm SL. Dorsal-fin rays XII, 9 (last ray composite); anal-fin rays III, 8 (last ray composite); pectoral-fin rays 15–16, modally 16, 16 on both sides of holotype; pelvic-fin rays I,5; total caudal-fin rays 25 (13 + 12), principal rays 17 (9 + 8), spinous procurrent rays 6 (III + III), and 2 additional rays (i + i) between principal and procurrent rays that are neither spinous nor typically segmented; vertebrae 25 (10 + 15); pattern of supraneural bones, anterior dorsal-fin pterygiophores, and dorsal-fin spines 0/0/0+2/1+1/1/; ribs on vertebrae 3–10; epineural bones present on at least vertebrae 1-14 in holotype; gill rakers on first arch 12-14 (3-4 + 8-10), modally 12 (3+9 or 4+8), 12 (3+9) in holotype; upper-limb rakers and lowermost one or two rakers very small or present only as nubs, all other gill rakers elongate and slender with tooth-like secondary rakers as in *L. evides* Robins & Colins 1979 (Baldwin et al [2016: fig. 3]); pseudobranchial filaments ~4–7 (~5 in holotype), filaments fat and fluffy but poorly formed in most specimens; branchiostegals 6.

**Table 1.** Counts and measurements of type specimens of *Lipogramma barrettorum* sp. n. Measurements are in percent SL. “Other Caudal Rays” include “i” – a slender, flexible, non-spinous, and typically non-segmented ray and “I” – a spinous procurrent ray.

	USNM 440439	USNM 406392	UF 239254	USNM 414914	USNM 431687	USNM 436460	USNM 436474
	Holotype	Paratype	Paratype	Paratype (juvenile)	Paratype	Paratype	Paratype (juvenile)
SL	26.5	24.5	28.0	13.0	25.2	27.0	10.2
Dorsal-fin Rays	XII, 9	XII, 9	XII, 9	XII, 9	XII, 9	XII, 9	XII, 9
Anal-fin Rays	III, 8	III, 8	III, 8	III, 8	III, 8	III, 8	III, 8
Principal Caudal Rays	9+8	9+8	9+8	9+8	9+8	9+8	9+8
Other Caudal Rays	IIIi+iIII	IIIi+iIII	IIIi+iIII	IIIi+iIII	IIIi+iIII	IIIi+iIII	IIIi+iIII
Pectoral-fin Rays	16, 16	15, 15	16, 15	15, 16	16, 16	16, 16	16, 15
Gill Rakers	3+9=12	3+10=13	4+10=14	-	3+~9=12	4+8=12	-
Head Length	39.6	36.7	38.2	39.2	40.1	38.9	39.2
Eye Diameter	13.2	13.1	12.1	14.6	12.7	12.6	14.7
Snout Length	7.9	7.3	8.2	7.7	7.9	8.9	8.8
Depth at Caudal Peduncle	19.2	18.4	21.8	17.7	17.5	20.4	18.6
Depth at Pelvic-fin Origin	31.3	31.4	30.0	29.2	31.7	30.4	31.4
Length of Pectoral Fin	24.9	24.5	Broken	Broken	22.2	22.6	19.6
Length of Pelvic Fin	49.1	42.9	Broken	31.5	34.5	40.0	34.3
Length of 12^th^ Dorsal Spine	18.5	20.8	19.6	15.4	17.9	18.9	19.6

Spinous and soft dorsal fins confluent, several soft rays at rear of fin forming slightly elevated lobe that extends posteriorly beyond base of caudal fin. Pelvic fin, when depressed, extending posteriorly to point between base of second or third anal-fin spine and posterior base of anal fin, first pelvic-fin ray elongate. Dorsal profile from snout to origin of dorsal fin convex. Diameter of eye of holotype contained three times in head length. Pupil slightly tear shaped, with small aphakic space anteriorly. Scales extending anteriorly onto posterior portion of head, ending short of coronal pore. Scales present on cheeks, opercle, preopercle, interopercle, and isthmus. Scales lacking on top of head, snout, jaws, and branchiostegals. Scales large and deciduous, too many scales missing in most specimens to make accurate scale counts. In one paratype (USNM 436460) approximately 21 lateral scales between shoulder and base of caudal fin, approximately 4 scale rows on cheek, and approximately 9 scale rows across body above anal-fin origin. Scales on head and nape without cteni, scales on rest of body ctenoid. Fins naked.

Margins of bones of opercular series smooth, opercle without spines. Single row of teeth on premaxilla posteriorly, broadening to 2–3 rows anteriorly, teeth in innermost row smallest, some teeth in outer row enlarged into small canines. Dentary similar, holotype with 6 enlarged teeth in outer row near symphysis. Vomer with chevron-shaped patch of teeth. Palatine with long series of small teeth. Conspicuous pores present in infraorbital canal (2 pores), portion of supraorbital canal bordering dorsal portion of orbit (4), on top of head (1 median coronal pore), preopercle (at least 5), and lateral-line canal in the post-temporal region (3). The 4 supraorbital pores situated as illustrated by Baldwin et al. (2016: fig 4) for *L. evides*. Posterior nostril situated just ventral to anteriormost supraorbital pore, nostril a single large opening. Anterior nostril at apex of elongate narial tube and situated just posterior to upper lip. No lateral line present on body.

Coloration: *In life or deceased but prior to preservation* (Fig. 1): ground color of body brownish yellow, head darker than trunk, especially on underside. *Head*: dorsal midline of head with thin blue-white stripe beginning on lower lip, continuing on upper lip and over snout to nape; iris yellow, blue-white bars anterior and posterior to pupil; an indistinct yellow/brown bar from center of lower edge of iris to lower jaw, bar bordered anteriorly and posteriorly by smaller pale bars that extend up to top of eye. *Trunk*: 9–12 narrow dark bars between pectoral-fin base and caudal peduncle, bars about as wide as paler interspaces. *Dorsal fin*: yellow, with a thin blue-grey margin; series of straight, wavy, or irregular blue-grey bars on basal 2/3 of spinous dorsal fin; basal half of soft-dorsal with a large black ocellus complete ringed in blue-white pigment, ocellus extending onto trunk; distal half of fin with 2–3 rows blue-grey round- to square-shaped spots, these markings (here and on other unpaired fins) with pale centers and darker edges. *Anal fin: y*ellow, with a thin blue-grey margin; 5–6 rows of blue-grey square-shaped spots between fin base and margin, some of which may fuse to form irregular lines. *Caudal fin*: yellow, with thin blue-grey posterior margin and 6–7 bars formed by vertical rows of blue-grey square-shaped spots on inter-radial membranes, basal two rows palest. *Pectoral fins*: translucent, base as dark as trunk bars. *Pelvic fins*: opposite color scheme to that of other fins, i.e., mostly blue-grey with yellow spots along inter-radial membranes; proximally, spots with tiny black center; distally, dark centers larger, some spots appearing completely dark. *Juveniles*: the 12- and 15-mm SL paratypes (Fig. 2) with similar pigment pattern as adults. *Comment regarding live coloration*: photographed against a light background (Fig. 1A, C), “blue-grey” in description above = grey; photographed against a black background (Fig. 1B), “blue-grey” = blue. *Preserved coloration* (Fig. 3A): Head mostly brown, trunk mostly tan with darker tan to brown bars. Yellow portions of median fins in life clear in preservative, blue-grey markings on fins in life dark brown to black in preservative.

#### Distribution.

Known only from specimens collected off Curaçao, southern Caribbean.

#### Habitat.

Lives in or immediately above elevated rocky habitat with ample cracks or holes into which the fish retreated upon approach of the submersible. The holotype was collected at 161 m, which is the only discrete depth recording for the species. Depth ranges for two specimens were recorded as 123–160 m and 132–141 m, thus providing a potential total depth range of 123–161 m. Depth ranges for two additional specimens of 90–249 m and 50–246 m reflect depths visited during an entire submersible dive and provide little information relevant to establishing this species’ depth distribution.

#### Etymology.

Named *Lipogramma barrettorum* in recognition of the support of Craig and Barbara Barrett for the Smithsonian’s Deep Reef Observation Project (DROP).

#### Common name.

We propose blue-spotted basslet in reference to the numerous blue/grey markings on the dorsal, anal, and caudal fins in life.

#### Genetic comparisons.

Table 2 shows average inter- and intraspecific divergences in COI among species of *Lipogramma* analyzed genetically in this study. Average intraspecific divergence among the seven specimens of *L. barrettorum* is 0.003 substitutions per site, and interspecific divergences between it and the other species for which data are available range from 9.9% (*L. evides*) to 25.3% (*L. klayi*).

**Table 2.** Average Kimura two-parameter distance summary for species of *Lipogramma* based on cytochrome c oxidase I (COI) sequences analyzed in this study. Intraspecific averages are in bold.

	*L. barretorum*	*L. regia*	*L. evides*	*L. schrieri*	*L. levinsoni*	*L. haberi*	*L. anabantoides*	*L. trilineata*	*L. klayi*
*L. barrettorum* (n=7)	**0.003**								
*L. regia* (n=1)	0.182	–							
*L. evides* (n=30)	0.099	0.201	**0.002**						
*L. schrieri* (n=7)	0.117	0.197	0.123	**0.002**					
*L. levinsoni* (n=15)	0.158	0.152	0.167	0.165	**0.001**				
*L. haberi* (n=3)	0.107	0.185	0.108	0.131	0.182	**0.002**			
*L. anabantoides* (n=2)	0.185	0.188	0.210	0.179	0.153	0.193	**0.005**		
*L. trilineata* (n=12)	0.214	0.207	0.242	0.246	0.227	0.235	0.254	**0.005**	
*L. klayi* (n=21)	0.253	0.243	0.253	0.253	0.248	0.264	0.239	0.240	**0.003**

#### Comments.

**Figure 4.**
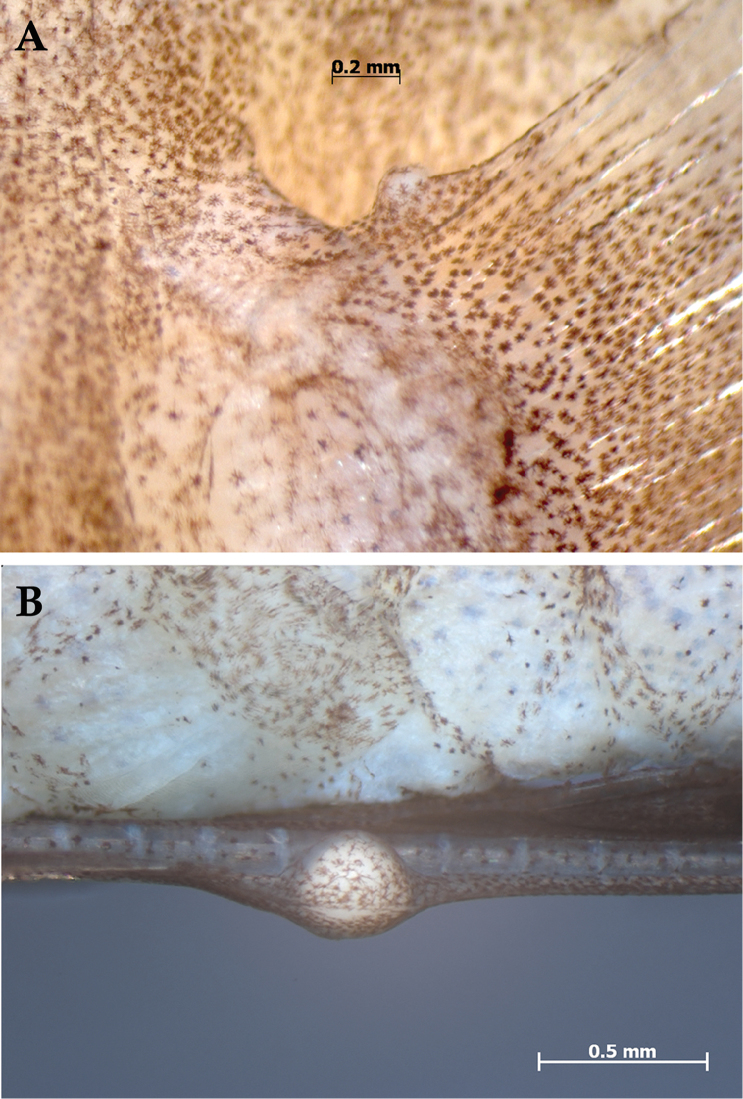
Cysts on fin rays of *Lipogramma barrettorum*, USNM 440439, CUR16008, holotype, 26.5 mm SL **A** left pectoral fin showing cyst on uppermost ray **B** elongate first left pelvic-fin ray showing cyst about midway along its length. Photographs by A. Nonaka.

The holotype has two cysts, one at the base of the uppermost left pectoral-fin ray and one about mid-way along the length of the elongate first left pelvic-fin ray (Fig. 4). The cysts or galls are likely parasitic, but further analysis is needed. No other cysts were observed on the holotype or paratypes.

### Lipogramma schrieri

Taxon classificationAnimaliaORDOFAMILIA

Baldwin, Nonaka & Robertson sp. n.

http://zoobank.org/4BB82A69-DE7F-438D-8DE3-740D00396C08

**Figure 5.**
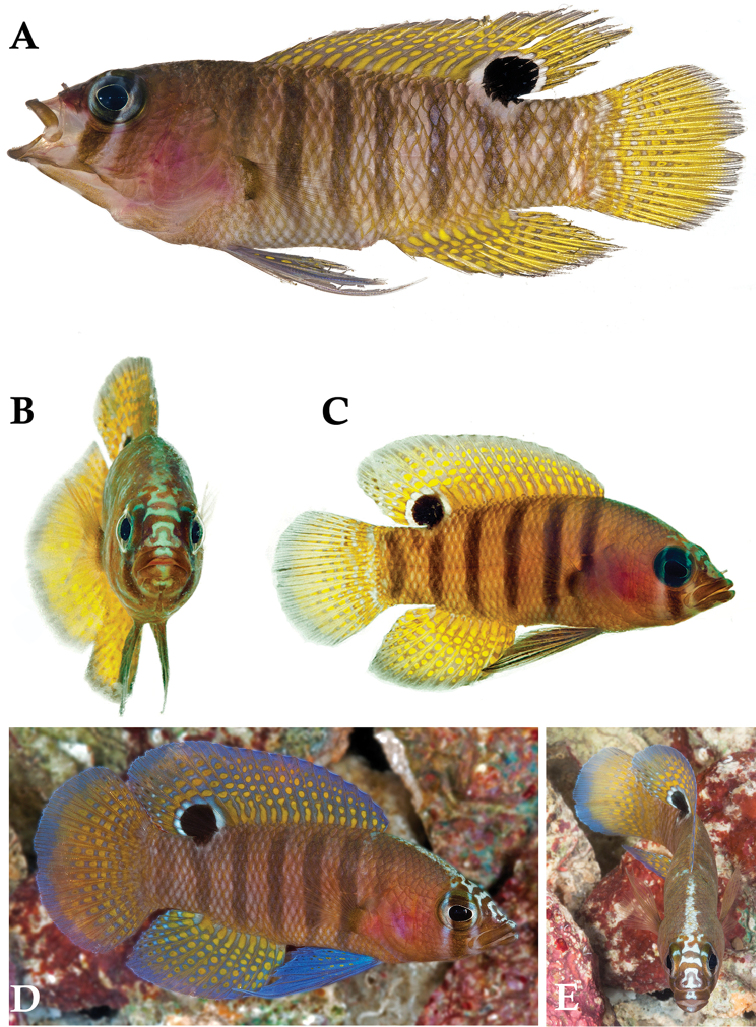
*Lipogramma schrieri* sp. n. **A** USNM 431722, CUR14114, holotype, 49.7 mm SL paratype, photograph by D. R. Robertson and C. C. Baldwin **B** and **C** specimen of unknown size collected off Curaçao (specimen not retained), aquarium photographs by Mac Stone **D** and **E** specimen of unknown size collected off Curaçao (specimen not retained), aquarium photographs by Barry Brown.

**Figure 6.**
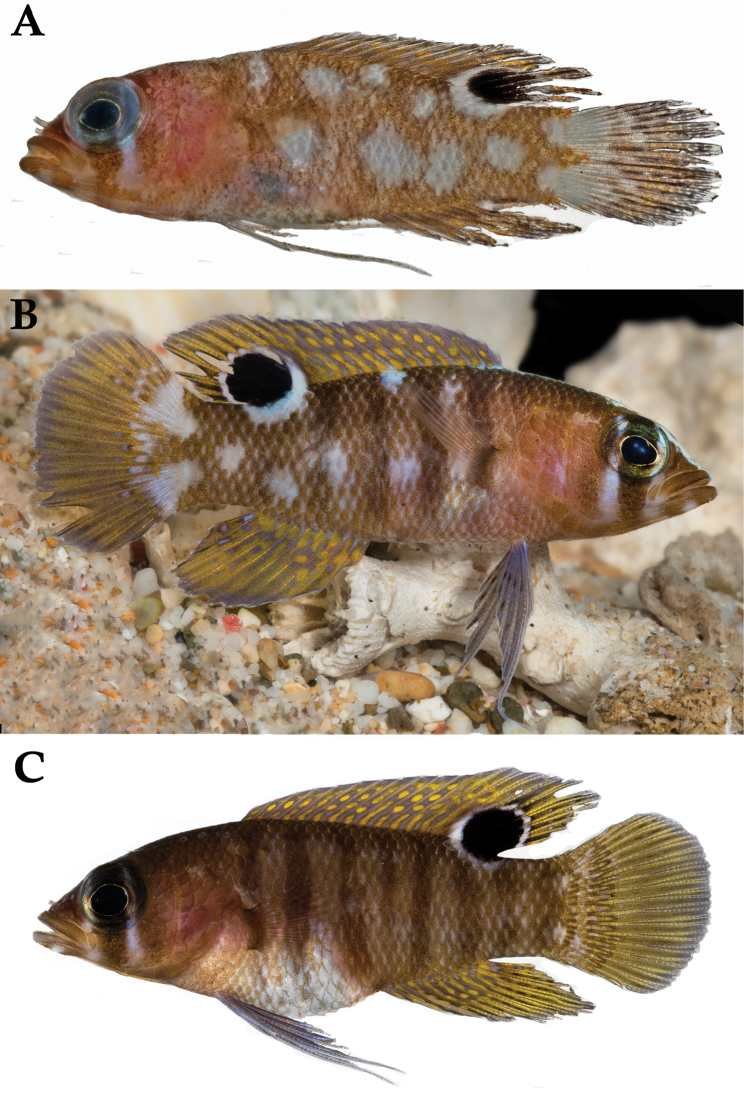
*Lipogramma schrieri* sp. n. **A** USNM 413864, CUR12290, paratype, 17.2 mm SL, photograph by D. R. Robertson and C. C. Baldwin **B** USNM 430035, CUR13329, paratype, 26.0 mm SL, photograph by Barry Brown **C** USNM 435299, CUR15012, paratype, 32.8 mm SL, D. R. Robertson and C. C. Baldwin.

English: Maori Basslet; Spanish: Cabrilleta maorí Figures 3 , 5 , 6

#### Type locality.

Curaçao, southern Caribbean.

**Holotype.** USNM 431722, 49.7 mm SL, tissue no. CUR14114, GenBank accession no. KX713790, Curasub submersible, sta. CURASUB 14-15, Curaçao, Jan Thiel Bay, 12.0746 N, 68.8825 W, 197 m, 19 September 2014, C. Baldwin, B. Brandt & A. Schrier.

**Paratypes.** USNM 414913, 56.0 mm SL, tissue no. CUR12101, Curasub submersible, sta. CURASUB12-12, Curaçao, east of Substation Curaçao downline, 12.0832 N, 68.8991 W, 156–290 m (no discrete depth observation), 7 August 2012, D. Pawson, B. Brandt, A. Schrier & C. Baldwin; USNM 414911, 61.9 mm SL, tissue no. CUR12316, Curasub submersible, sta. CURASUB12-MISC, Curaçao, off Substation Curaçao, 12.0832 N, 68.8991 W, no depth data, 21 May 2012, Substation Curaçao staff; UF 239255, 46.6 mm SL, tissue no. CUR12317, same collection information as USNM 414911; USNM 430035, 26.0 mm SL, tissue no. CUR13329, Curasub submersible, sta. CURASUB 13-31, Curaçao, west of Substation Curaçao downline, 12.0832 N, 68.8991 W, 177 m depth, 1 November 2013, C. Baldwin, B. Brandt, R. Robertson & C. Castillo; USNM 435299, 32.8 mm SL, tissue no. CUR15012, Curasub submersible, sta. CURASUB15-05, Curaçao, east of Substation Curacao downline, 12.0832 N, 68.8991 W, 173 m depth, 10 February 2015, C. Baldwin, B. Brandt, R. Robertson & C. Castillo; USNM 413864, 17.2 mm SL, tissue no. CUR12290, Curasub submersible, sta. CURASUB12-18, Curaçao, off Substation Curaçao, 12.0832 N, 68.8991 W, 207 m, 14 August 2012, C. Baldwin, B. Brandt, A. Schrier & A. Driskell.

#### Diagnosis.

A species of *Lipogramma* distinguishable from congeners by the following combination of characters: pectoral-fin rays 16-17 (modally 16), gill rakers 11–13 (modally 12, 8–9 rakers on lower limb); four supraorbital pores present along dorsal margin of orbit, a pore present between one above mid orbit and one above posterodorsal corner of orbit; caudal fin rounded; body mostly tan to brown in life with 7 or 8 narrow darker brown bars on trunk; head with broad, irregular, whitish blue markings along dorsal midline from lower lip across upper lip and snout to nape; dark bar through eye bordered anteriorly and posteriorly by bluish-white bars; posterior base of soft dorsal fin with large white- or blue-rimmed black ocellus; dorsal and anal fins blue-grey with yellow spots or bars. Caudal fin mostly yellow with wide blue-grey margin and several bars comprising blue-grey mostly square-shaped spots. Pelvic fins grey/blue with scattered yellow-ringed dark spots. Juveniles with irregular white blotches of pigment on trunk and two triangular white blotches on caudal-fin base. The new species is further differentiated genetically from congeners for which molecular data are available in mitochondrial COI and nuclear Histone 3, Rhodopsin, TMO-4C4, and RAG1.

#### Description.

Counts and measurements of type specimens given in Table 3. Seven specimens examined, 17.2–61.9 mm SL. Dorsal-fin rays XII, 9 (last ray composite); largest specimen (USNM 414911) with 9 pterygiophores in soft anal fin, but only 8 externally visible rays, the 8^th^ appearing to represent fusion of two rays; anal-fin rays III, 8 (last ray composite); pectoral-fin rays 16–17, modally 16, 16 on both sides in holotype; pelvic-fin rays I,5; total caudal-fin rays 25 (13 + 12), principal rays 17 (9 + 8), spinous procurrent rays 6 (III + III), and 2 additional rays (i + i) between principal and procurrent rays that are neither spinous nor typically segmented; vertebrae 25 (10 + 15); pattern of supraneural bones, anterior dorsal-fin pterygiophores, and dorsal-fin spines usually 0/0/0+2/1+1/1/, one paratype (USNM 435299) aberrant in having first two dorsal-fin spines supported in supernumerary association by separate pterygiophores vs. a single pterygiophore – 0/0/0+1+1/1+1/1/; ribs on vertebrae 3–10; epineural bones present on at least vertebrae 1–15 in holotype and two paratypes, difficult to assess in other specimens; gill rakers on first arch 11–13 (3–4 + 8–9), 11 (3 + 8) in holotype; upper-limb rakers and lowermost one or two rakers very small or present only as nubs, all other gill rakers elongate and slender with tooth-like secondary rakers as in *L. evides* (Baldwin et al. [2016: fig 3]); pseudobranchial filaments 7–11 (9 in holotype), filaments poorly or well developed (well developed in holotype); branchiostegals 6.

**Table 3.** Counts and measurements of type specimens of *Lipogramma schrieri* sp. n. Measurements are in percent SL. “Other Caudal Rays” include “i” – a slender, flexible, non-spinous, and typically non-segmented ray and “I” – a spinous procurrent ray.

	USNM 431722	USNM 413864	USNM 414913	USNM 414911	UF 239255	USNM 430035	USNM 435299
	Holotype	Paratype (juvenile)	Paratype	Paratype	Paratype	Paratype	Paratype
SL	49.7	17.2	56.0	61.9	46.6	26.0	32.8
Dorsal-fin Rays	XII, 9	XII, 9	XII, 9	XII, 9	XII, 9	XII, 9	XII, 9
Anal-fin Rays	III, 8	III, 8	III, 8	III, 8	III, 8	III, 8	III, 8
Principal Caudal Rays	9+8	9+8	9+8	9+8	9+8	9+8	9+8
Other Caudal Rays	IIIi+iIII	IIIi+iIII	IIIi+iIII	IIIi+iIII	IIIi+iIII	IIIi+iIII	IIIi+iIII
Pectoral-fin Rays	16, 16	16, 16	16, -	16, 16	16, 16	17, 17	16, 16
Gill Rakers	3+8=11	-	3+9=12	4+9=13	3+9=12	4+9=13	3+9=12
Head Length	38.6	36.1	33.9	36.0	37.6	37.3	38.7
Eye Diameter	11.7	15.1	11.4	12.1	10.9	13.5	14.0
Snout Length	10.1	6.4	8.8	9.1	9.0	8.5	7.9
Depth at Caudal Peduncle	19.7	19.8	20.4	21.0	20.2	20.0	19.2
Depth at Pelvic-fin Origin	32.8	31.4	31.8	31.8	32.2	32.3	31.4
Length of Pectoral Fin	20.9	21.5	20.9	22.9	Broken	22.7	21.7
Length of Pelvic Fin	Broken	39.5	40.4	40.6	Broken	39.6	39.0
Length of 12^th^ Dorsal Spine	15.7	19.2	23.6	18.9	19.7	22.3	20.7

Spinous and soft dorsal fins confluent, several soft rays in posterior portion of fin forming slightly elevated lobe that extends posteriorly beyond base of caudal fin. Pelvic fin extending posteriorly to base of third anal-fin spine in preserved holotype when depressed, to middle or posterior portion of anal fin in aquarium photos (e.g., Fig. 5D). Dorsal profile from snout to origin of dorsal fin convex. Diameter of eye of holotype contained 3.3 times in head length. Pupil slightly tear shaped with small aphakic space anteriorly. Scales extending anteriorly onto top of head, ending short of coronal pore. Scales present on cheeks, operculum, and isthmus. Scales lacking on frontal region, snout, jaws, and branchiostegals. Scales large and deciduous, too many missing in most preserved specimens to make counts, but counts made from photographs of specimens prior to preservation indicate approximately 25–27 lateral scales between shoulder and base of caudal fin (27 in holotype), 5 cheek rows, and 12 rows across body above anal-fin origin. Scales on head and nape without cteni, scales on rest of body ctenoid. Fins naked.

Margins of bones of opercular series smooth, opercle without spines. Premaxilla with band of small conical teeth, band widest at symphysis, outer row with largest teeth, 3 or 4 (4 in holotype) near symphysis enlarged. Dentary similar except 8 anterior teeth enlarge. Vomer with chevron-shaped patch of teeth, palatine with long series of small teeth. Conspicuous pores present in infraorbital canal (2 pores), portion of supraorbital canal bordering dorsal portion of orbit (4), on top of head (1 median coronal pore), preopercle (at least 5), and lateral-line canal in the posttemporal region (3). The 4 supraorbital pores situated as illustrated by Baldwin et al. (2016: fig 4) for *L. evides*. Posterior nostril situated just ventral to anteriormost supraorbital pore, nostril a single large opening. Anterior nostril at apex of elongate narial tube and situated just posterior to upper lip. No lateral line present on body.

Coloration: *In life or deceased but prior to preservation* (Fig. 5), ground color of body light brown. *Head*: dorsal midline of head with broad area of irregular, blue-white markings beginning on lower lip and continuing on upper lip and over snout to nape; a dark brown, pupil-width bar extending across orbit to lower jaw, this bar bordered on either side by thin whitish bar that runs from top of eye through front and rear of iris to lower jaw. *Trunk*: 7–8 narrow, dark-brown bars between posterior edge of operculum and caudal peduncle, bars narrower than paler interspaces. *Dorsal fin*: spinous dorsal and anterior portion of soft dorsal blue-grey with stripes comprising short yellow bars proximally and yellow spots distally; posterior portion of soft dorsal with large black ocellus ringed in blue-white pigment that extends onto trunk; several rows of yellow spots above ocellus. *Anal fin*: blue-grey, with yellow markings similar to those on spinous dorsal fin. *Caudal fin*: yellow, with vertical bars comprising blue-grey, square-shaped spots on inter-radial membranes on anterior 2/3 of fin; wide, blue-grey margin distally. *Pectoral fins*: most of fin translucent, base and anterior portion of fin dark. *Pelvic fins*: pale blue-grey to bright blue with yellow-ringed dark spots, spots mostly dark brown distally. *Juveniles*: An ontogenetic series from 17–33 mm SL is shown in Figure 6. The 17-mm SL specimen lacking body bars and with row of four large, irregular white blotches on or just below lateral midline of trunk, smaller white spots along back above that row, white spot at posterior base of anal fin, and two large, roughly triangular white blotches on caudal-fin base. First four dark trunk bars evident anteriorly in 26-mm SL juvenile, which has smaller white markings (spots vs. blotches). In 33-mm SL specimen, all trunk bars present, and remnants of each white caudal-fin blotch present as small white spot before indistinct pale vertical bar. *Comment regarding live coloration*: photographed against a light background (Fig. 5A–C), “blue-grey” in description above = grey; photographed against a darker background (Fig. 5D, E), “blue-grey” = blue. *Preserved coloration* (Fig. 3B): Head and trunk tan with darker tan to brown bars. Yellow portions of median fins in life clear in preservative, blue-grey markings on fins in life dark brown in preservative.

**Distribution.** Known only from specimens collected off Curaçao, southern Caribbean.

#### Habitat.

Elevated rocky habitat with ample cracks or holes into which the fish retreated upon approach of the submersible. The holotype was collected at 197 m, and three paratypes were collected at 173–207 m. The range of 156–290 m recorded for another paratype reflects all depths visited on the submersible dive during which the specimen was collected and provides little relevant depth information.

#### Etymology.

Named *Lipogramma schrieri* in honor of Adriaan (Dutch) Schrier, owner of Substation Curaçao. Although the *Curasub* submersible was not built originally for scientific research, Dutch’s enthusiastic support of research use of his sub has exponentially expanded our understanding of fish and invertebrate faunas of Caribbean mesophotic and deeper reefs.

#### Common name.

We propose Maori Basslet, in reference to the similarity of the markings on the dorsal midline of the forehead to the beautiful facial tattoo of the Maoris, indigenous Polynesian people of New Zealand.

#### Genetic Comparisons.

Table 2 shows average inter- and intraspecific divergences in COI among species of *Lipogramma* analyzed genetically in this study. Average intraspecific divergence among the seven specimens of *L. schrieri* is 0.002 substitutions per site, and interspecific divergences between it and the other species for which data are available range from 11.7% (*L. barrettorum*) to 25.3% (*L. klayi*).

#### Comments.

**Figure 7.**
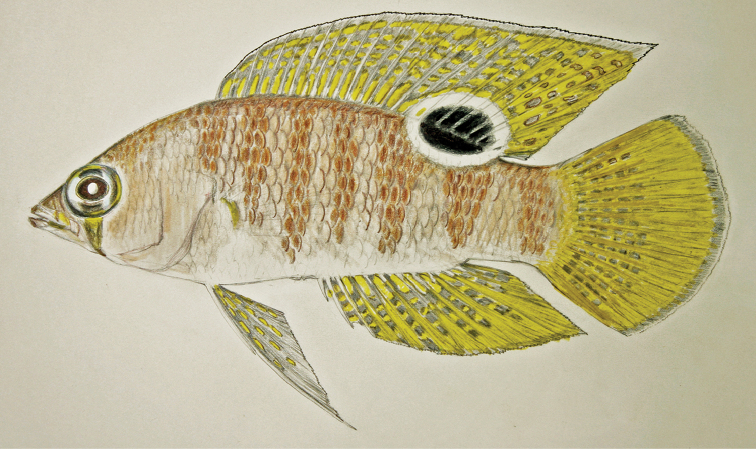
*Lipogramma* sp. from Cuba, RGG uncataloged, 52.5 mm SL, collected at Cayos Los indios, JSLII Dive 3069, 296 m, 27 Dec 1997, R. G. Gilmore and R. Robins. Drawing by R. G. Gilmore.

A 52.5 mm SL *Lipogramma* (RGG uncataloged), collected at 296 m in 1997 by one of us (RGG) and Richard Robins off Cuba (Fig. 7), could be a specimen of *L. schrieri.* It has a similar color pattern, including seven dark body bars, the “maori” pattern of pigment on top of the head (based on examination of the preserved specimen), and similar pattern of fin pigment. However, this specimen has yellow pigment around the eye (vs. brown in *L. schrieri*), yellow pigment in a triangular-shaped subocular bar (vs. brown pigment in a rectangular-shaped bar), and a yellow pectoral-fin base (vs. dark brown). Furthermore, the Cuban specimen has 15 pectoral-fin rays on each side vs. 16–17 (modally 16) in *L. schrieri*, and 14 total gill rakers vs. 11–13 in *L. schrieri*. Further study is needed to determine if this specimen represents a variant of *L. schrieri* or an additional cryptic species in the genus.

## Discussion

**Morphological comparisons.** *Lipogramma barrettorum, L. robinsi*, and *L. schrieri* differ from all congeners in having at least seven dark trunk bars. A comparison of major morphological and pigmentation differences among those three species is provided in Table 4. They are easily separable from one another on the basis of live or fresh color patterns, most notably the following: (1) the presence of 7–8 dark brown trunk bars against a tan background in *L. schrieri*, 10–12 tan/yellow bars against a flesh to greenish background in *L. robinsi*, and 11–12 light brown bars against a yellow background in *L. barrettorum* (trunk bars also evident in preserved specimens); (2) the presence of a bright yellow nape in *L. robinsi*, a blue-white stripe from the tip of the lower jaw to the base of the dorsal fin in *L. barrettorum*, and irregular, broad, blue-white markings from the tip of the lower jaw to the nape in *L. schrieri* (stripe in *L. barrettorum* and irregular markings in *L. schrieri* also evident in preserved specimens); (3) the presence of a distinct dark bar through the eye in *L. schrieri*, no such bar in *L. robinsi*, and an indistinct bar in *L. barrettorum* (orbital bar, when present, also evident in preserved specimens); and (4) median fins transparent with yellow spots in *L. robinsi*, yellow with blue/grey spots in *L. barrettorum*, and blue/grey with yellow spots in *L. schrieri.Lipogramma robinsi* can further be distinguished from the other two species by having more lower-limb gill rakers on the first arch (11–12 vs. 8–9 in *L. schrieri* and 8–10 in *L. barrettorum*) and usually having fewer pectoral-fin rays (15 vs. modally 16 in *L. schrieri* and *L. barrettorum*). Based on available material, *Lipogramma schrieri* reaches a larger size (~62 mm SL) than *L. robinsi* (~22 mm SL) and *L. barrettorum* (~25 mm SL).

**Table 4.** Summary of major morphological and pigmentation differences among *Lipogramma robinsi, L. barrettorum* sp. n., and *L. schrieri* sp. n.

ADULT	*L. robinsi*	*L. barrettorum*	*L. schrieri*
	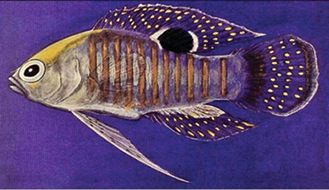	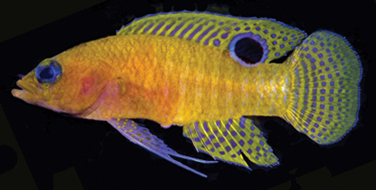	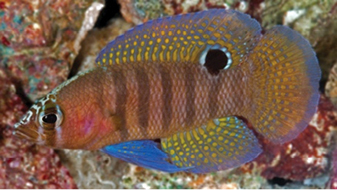
SL in preservative	To 22 mm	To 25 mm	To 62 mm
Gill Rakers	14–16, 11–12 on lower limb	12 (12–14), 8–10 on lower limb	12 (11–13), 8–9 on lower limb
Pectoral-fin rays	15	16 (15–16)	16 (16–17)
Body ground color	Translucent green to flesh	Yellow to yellowish brown	Tan/brown
Head coloration in life	Grey-brown; top of head bright yellow without blue-white marksDark bar through eye to mouth absent	Yellow-brown; top of head with median blue-white stripeDark bar through eye to mouth indistinct	Pale brown; top of head with blue-white “tattoo”marksDark bar through eye to mouth strong
Trunk in life	10–12 narrow yellow bars, narrow interspaces	11–12 narrow brown bars, narrow interspaces	7–8 narrow brown bars, wide interspaces
Dorsal Fin in life	Transparent with yellow spots; margin whiteSoft dorsal: black ocellus with white front & rear edges	Yellow with blue-grey wavy bars and spots; margin grey-blueSoft dorsal: black ocellus ringed with blue-white	Blue-grey with yellow spots and short bars; margin blue-greySoft dorsal: black ocellus ringed with blue-white
Anal fin in life	Translucent; base white; rows yellow spots	Yellow with blue-grey spots and wavy lines; margin blue-grey	Blue-grey with yellow spots and bars; margin blue-grey
Caudal Fin in life	Translucent; base yellow, center with yellow spots, margin white	Yellow with bars of blue-grey spots; blue-grey margin.	Yellow with bars of blue-grey spots; margin blue-grey
Pectoral fins in life	Translucent	Translucent	Translucent; base dark
Pelvic fins in life	White; rows black spots	Blue-grey with yellow-ringed dark spots	Pale blue-grey to blue with yellow-ringed dark spots
**JUVENILE**	Not known	Known from 10–13 mm (preserved SL) specimens	Known from 17–33 mm (preserved SL) specimens
Trunk	Not known	Similar to adult	Scattered white spots and blotches on trunk and base of caudal fin, blotches roughly in two rows of four in smallest juvenile (17 mm SL); anterior trunk bars first evident in 26-mm SL juvenile paratype
Anal fin	Not known	Similar to adult	Similar to adult except more yellow distally
Caudal fin	Not known	Similar to adult	Mostly yellow with blue-grey margin and 2 large triangular white blotches on base

**Figure 8.**
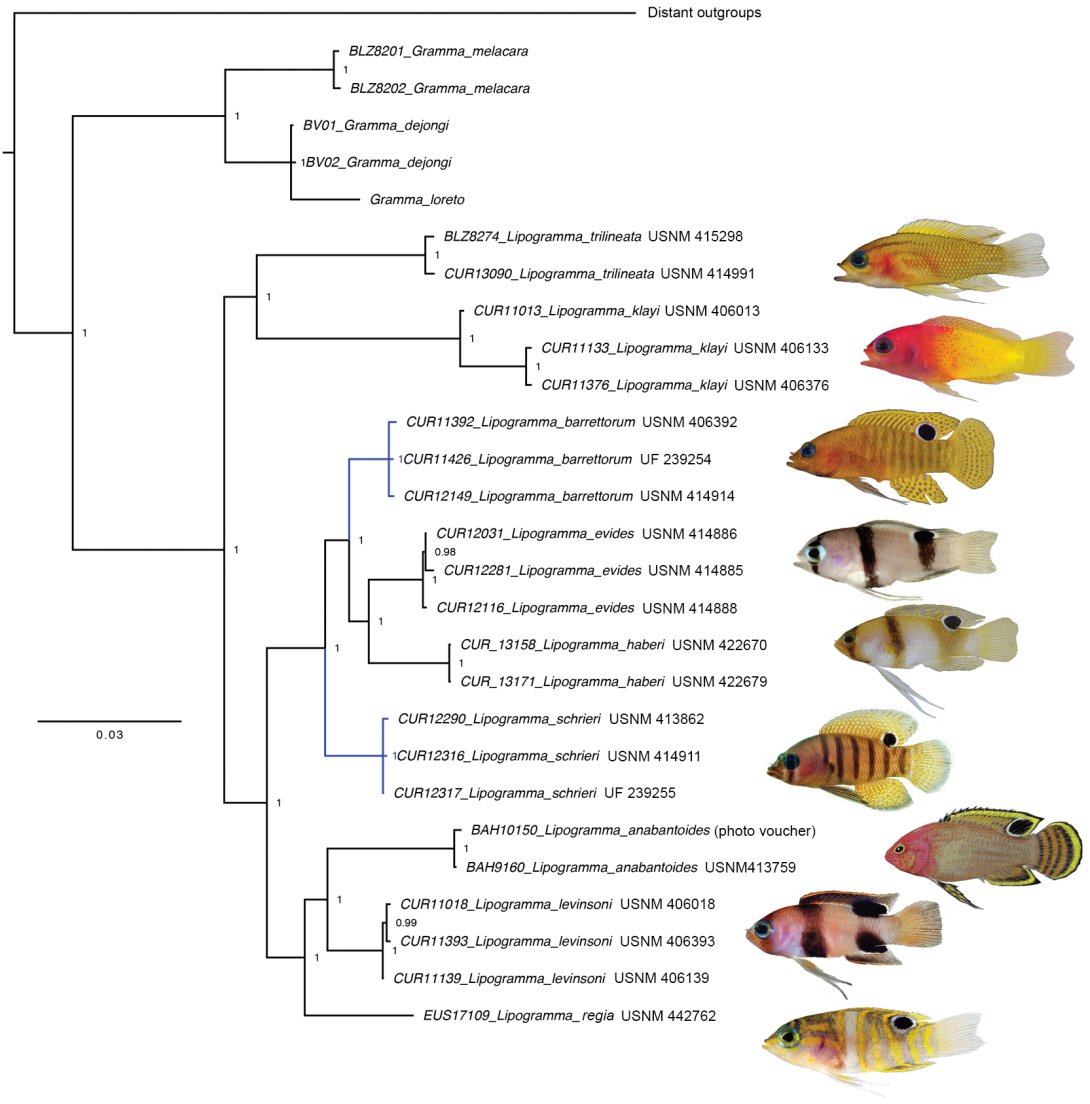
Bayesian Inference molecular phylogeny of nine species of *Lipogramma* based on combined mitochondrial and nuclear genes. Numbers of individuals analyzed for each species are given in Appendix 1, along with the genes sequences for each individual. Topology is identical to that from BP&P species-tree analysis. Support values are Bayesian posterior probabilities (above) and bootstrap values (below). Nodes without labels have 1.0 posterior probability and 100 bootstrap values. Photographs or illustrations by C. C. Baldwin, R. G. Gilmore, D. R. Robertson, C. R. Robins, and M. Stone.

**Species delimitation and phylogeny.** Comparative morphological analysis supports the recognition of *L. barrettorum* and *L. schrieri* as distinct species, and combinations of diagnostic morphological features that distinguish them from all other *Lipogramma* species are provided in the species descriptions above. Molecular data for the nine *Lipogramma* species for which genetic data existed prior to this study (Baldwin et al. 2016) or was generated in this study (*L. anabantoides, L. barrettorum, L. evides, L. haberi, L. klayi, L. levinsoni, L. regia, L. schrieri, L. trilineata*) unequivocally support the presence of nine species (molecular data not available for *L. flavescens, L. roseum* and *L. robinsi*). The neighbor-joining network (Suppl. Material 1) derived from COI data shows nine distinct lineages with very high genetic distances between lineages (10–27%, mean = 19%), which are at least 20 times greater than variation in COI within lineages (range 0.1–0.5%, mean = 0.3%). The molecular phylogeny from the Bayesian analysis of the concatenated dataset (Fig. 8) was identical in topology to the BP&P coalescent-based species-tree analysis. The BP&P analysis also had overwhelming support for a nine-species model (posterior probability 0.996) versus models with fewer or more species.

**Figure 9.**
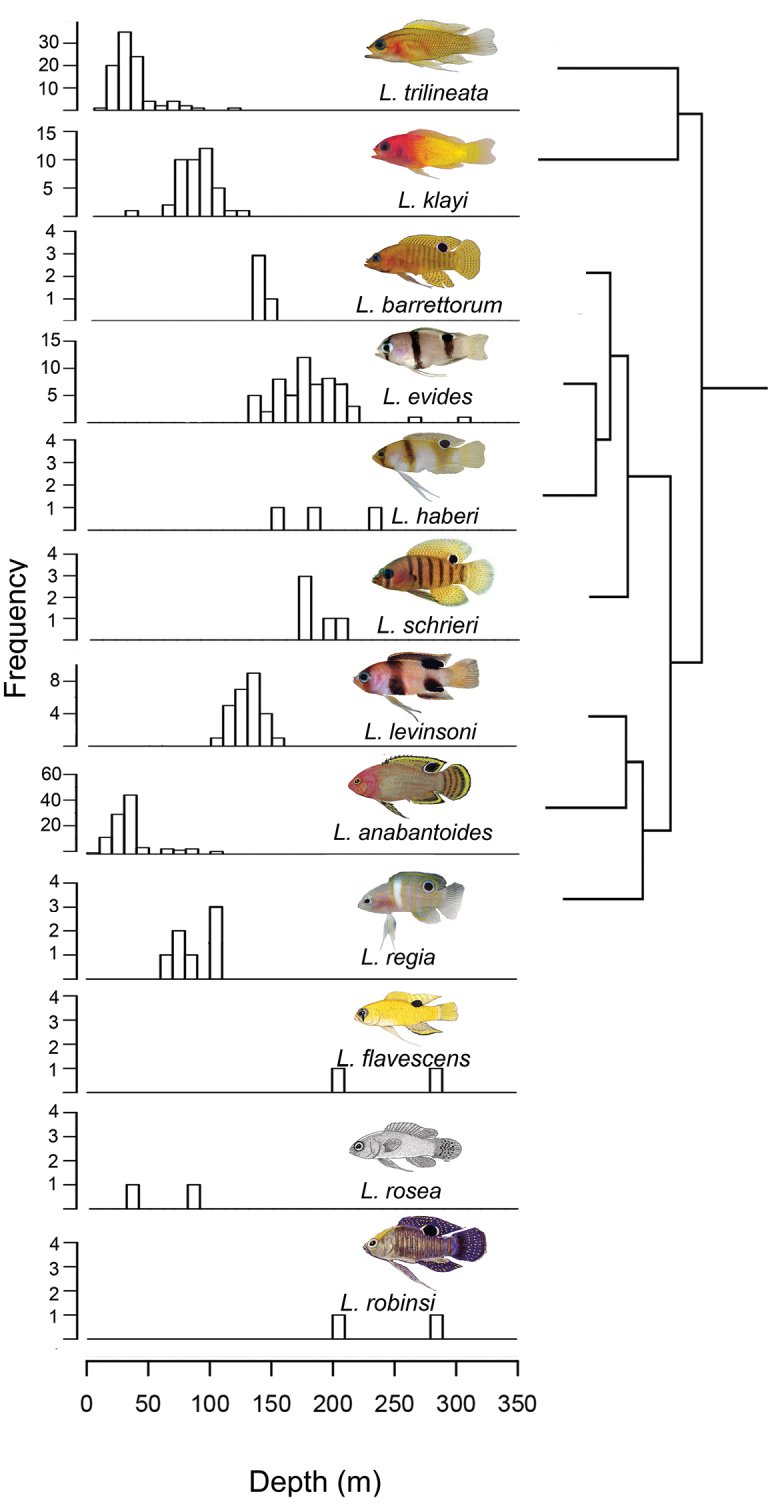
Eco-evolutionary histogram for species of *Lipogramma* showing phylogenetic distribution of species’ depth ranges. Photographs or illustrations by C. C. Baldwin, R. G. Gilmore, Gilmore (1997: fig. 1), Mooi and Gill (2002: fig. 9), D. R. Robertson, and M. Stone.

**Eco-evolutionary relationships.** The two new species, *L. barrettorum* and *L. schrieri*, belong to a clade that includes *L. evides* and *L. haberi*. Collectively the members of this clade are the deepest-living species in our analysis, occurring at depths predominantly below 140 m (Figure 9). Sister to this clade is a group comprising *L. levinsoni*, *L. anabantoides*, and *L. regia*, species that inhabit depths predominantly shallower than 150 m, in the zone traditionally referred to as mesophotic coral ecosystems (MCEs—~30-150 m; Kahng et al. 2010). A second shallow/MCE (< 150 m) clade that is sister to both those clades comprises *L. trilineata* and *L. klayi.* There is clear habitat partitioning by depth within both shallow clades, particularly between sister species: *L. trilineata* vs *L. klayi* and *L levinsoni* vs *L. anabantoides*. Based on available data, there is no clear depth partitioning evident within the more speciose *L evides* clade. We note, however, that these depth distributions are based on collections and observations of different species from different locations. Hence, we cannot rule out the possibility that some of the differences in depth distributions in Figure 9 represent location effects rather than depth partitioning.

In addition to depth, we have observed distinct interspecific variation in the types of substrata with which some of these species associate and the nature of their associations with substrata. Off Curaçao, *Lipogramma klayi* is a very commonly observed species, especially on the upper level of vertical faces or slopes of ~30-60° that are heavily indented with small caves and overhangs and festooned with gorgonians and other growth. We have commonly seen it occupying the same habitat at Bonaire, Dominica, Roatan, and St. Eustatius. Its sister species, *L. trilineata* is much more cryptic than *L. klayi.* It is rarely observed, as it tends to stay close to ceilings of cavities, whether those are caves or small holes formed in large rock or coral heads. Within the clade comprising the two new species, *L. evides* and *L. levinsoni* are commonly associated with small patches of cobble scattered among rocky areas, whereas *L. barrettorum* and *L. schrieri* are associated with elevated rocky habitat with ample cracks or holes. Finally, two of us (LT and DRR) recently observed multiple instances of *L. flavescens* off Roatan sitting on coarse sand, meters away from shelter, in areas of sand and scattered small low patches of rock.

Baldwin et al. (2016) noted that members of the large clade comprising all *Lipogramma* species except *L. trilineata* and *L. klayi* are characterized by a dark ocellus on the posterior base of the dorsal fin. Based on this character, a relatively shallow depth range, and a modal count of 17 rays in the pectoral fin, they hypothesized that *L. regia* (not sampled in that study) is most closely related to *L. anabantoides* and *L. levinsoni*. The present phylogenetic analysis includes a recently collected specimen of *L. regia* and supports this hypothesis. Baldwin et al. (2016) also hypothesized that *L. flavescens* may be part of the deep-dwelling clade comprising *L. evides*, *L. haberi*, *L. barrettorum*, and *L. schrieri* (the last two as “*Lipogramma ‘robinsi*’” in their phylogeny) and, based on the deep depth-range of *L. flavescens*, a dark ocellus on the dorsal fin, bright yellow body coloration, a dark bar through the eye, and a low gill-raker count (15–16), possibly most closely related to *L. haberi.* Collection of fresh material of *L. flavescens* would provide the genetic material needed to test their hypotheses. We note that *L. robinsi* is likely part of this deep-dwelling clade as well, based on the presence of a dorsal-fin ocellus, a barring pattern on the body similar to that of *L. schrieri* and *L. barrettorum*, and its depth range (although the latter is based on very few specimens). Fresh material of *L. robinsi* for genetic analyses is also desirable. In addition, more information is needed on the depth distributions of all of the less common species, particularly to determine the extent of habitat partitioning within locations, as well as its consistency between locations.

**Submersible exploration.** Effective capture of fish specimens during deep-sea submersible dives has only been realized since 1982 with the *Johnson Sea-Link* submersibles and, much more recently, with the *Curasub*. Fresh and often living specimens are brought to the surface, providing quality material for color photography and genetic analyses to investigate phylogenetic relationships and evolutionary trends. Capture of cryptobenthic species, including gobiids (Baldwin and Robertson 2015; Tornabene et al. 2016a, 2016b; Tornabene and Baldwin 2017), blennioids (Baldwin and Robertson 2013), grammatids (Gilmore and Jones 1988; Gilmore 1997; Baldwin et al. 2016b; this study), serranids (Baldwin and Johnson 2014, Baldwin and Robertson 2014), and scorpaenids (Baldwin et al. 2016a) using manned submersibles is allowing unprecedented examination of microhabitat relationships, depth and temperature preferences, and biogeography, along with comparative morphology and molecular phylogenetic relationships in previously unknown or inaccessible species. We cannot overemphasize the value of these manned undersea operations to increasing our knowledge and understanding of tropical deep-reef fish assemblages.

### Revised key to the species of *Lipogramma* (modified from Mooi and Gill 2002)

Photographs or illustrations by C. C. Baldwin, R. G. Gilmore, Gilmore (1997: fig. 1), Mooi and Gill (2002: fig. 9), D. R. Robertson, and M. Stone.

**Table:** 

1	Posterior base of soft dorsal fin with prominent dark spot, ocellus, or elongate blotch	**2**
–	Soft dorsal fin without prominent markings	**10**
2	With prominent black bar through eye	**3**
–	Without prominent black bar through eye	**7**
3	Trunk without bars, body yellow above, white below	***flavescens***
	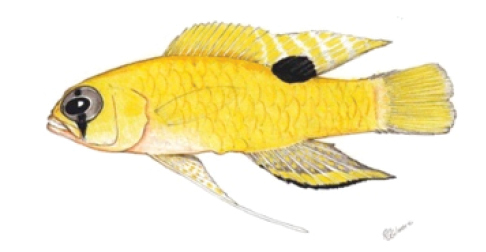	
–	Trunk with bars	**4**
4	Trunk with 2 bars	**5**
–	Trunk with 7–8 bars	***schrieri***
	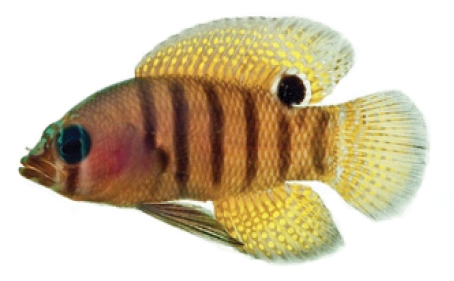	
5	Bar through eye wide, encompassing entire eye; trunk bars of equal intensity, often hourglass shaped; pectoral-fin rays modally 17, gill rakers modally 19	***levinsoni***
	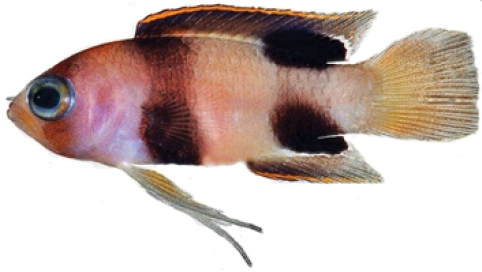	
–	Bar through eye narrow, encompassing only pupil; anterior trunk bar more pronounced than posterior bar, neither bar hourglass shaped; pectoral-fin rays modally 16, gill rakers 15–16 or 20–21	**6**
6	Posterior trunk bar a broad, yellowish inverted triangle; gill rakers modally 15–16	***haberi***
	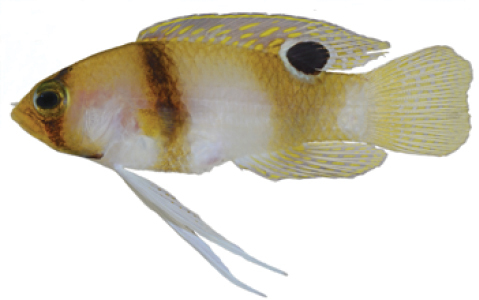	
–	Posterior trunk bar a black rectangle; gill rakers modally 20–21	***evides***
	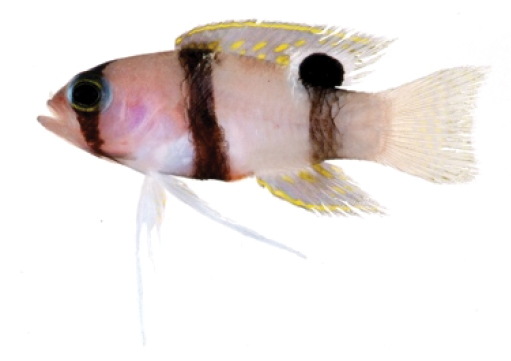	
7	Trunk without bars; head reddish, trunk grey-brown	***anabantoides***
	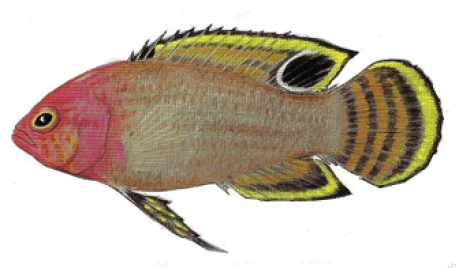	
–	Trunk with bars; head and trunk not colored as above	**8**
8	Trunk with 6 yellow bars, anterior 5 extending onto dorsal fin and 3^rd^-5^th^ extending onto anal fin; head with two prominent broad yellow stripes behind eye; top of head without yellow cap	***regia***
	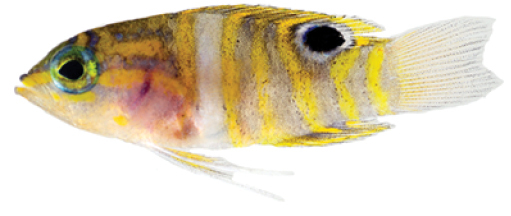	
–	Trunk with 10 to 12 bars, none extending onto dorsal or anal fins; head without broad yellow stripes behind eye, and top of head with or without yellow cap	**9**
9	Median fins transparent with yellow spots; top of head with yellow cap, without median blue-white stripe; lower-limb gill rakers 11–12	***robinsi***
	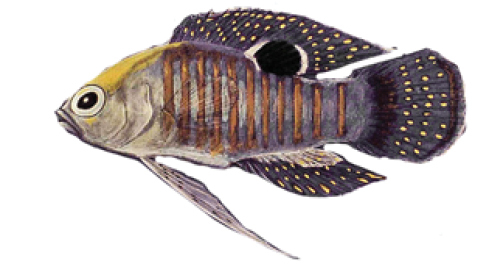	
–	Median fins yellow with blue-grey spots; top of head without yellow cap, with median blue-white stripe; lower-limb gill rakers 8–10	***barrettorum***
	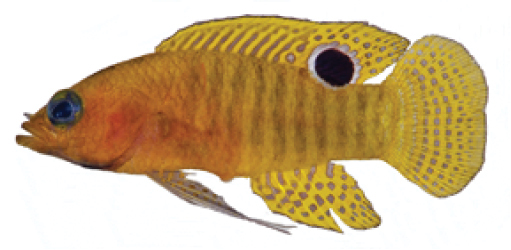	
10	Dorsal fin XI, 6–7; circum-peduncular scales 16; head and body yellow to rose colored, caudal fin yellow with dark spots, dorsal and anal fins red with pale spots	***rosea***
	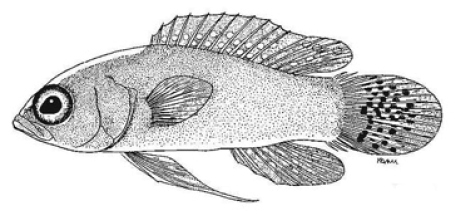	
–	Dorsal fin XII-XIII, 8–10; circum-peduncular scales 18 to 21; not colored as above	**11**
11	Strongly bicolored, purplish red anteriorly, yellow posteriorly; no stripes on head; scales in lateral series 29 to 35; gill rakers 20 to 21; anal-fin soft rays 8; upper caudal-fin spines 4 or 5	***klayi***
	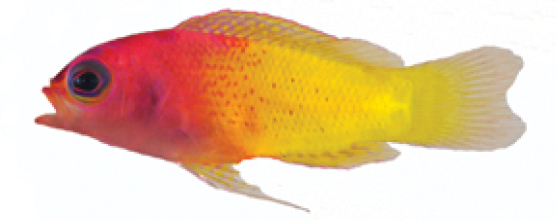	
–	Uniformly yellowish, 3 blue stripes on head: one along dorsal midline from snout to dorsal-fin, one from top of each eye to shoulder and anterior portion of trunk; lateral scales 25 to 29; gill rakers 13 to 18; anal-fin soft rays 7; upper caudal-fin spines 3	***trilineata***
	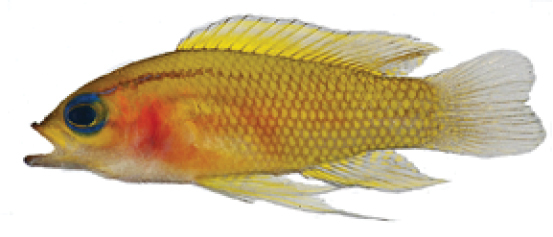	

## Supplementary Material

XML Treatment for Lipogramma barrettorum

XML Treatment for Lipogramma schrieri

## Appendix 1

**Table:** Links between DNA voucher specimens, GenBank accession numbers, and DNA sequences of Lipogramma derived for use in this study. BAH = Bahamas, BLZ = Belize, CUR = Curaçao, DOM = Dominica, EUS = St. Eustatius, T1K = Curaçao.

CatalogNumber	TissueNumber	Species	GenBank COI	GenBank H3	GenBank TMO-4C4	GenBank Rag1	GenBank Rhodopsin	GenSeq designation
Photo Voucher Only	BAH10150	*Lipogramma anabantoides*	KX713732	KX713823	KX713880	KX713842	KX713862	genseq-5
USNM 413759	BAH9160	*Lipogramma anabantoides*		KX713824	KX713881	KX713843	KX713863	genseq-4
USNM 420334	BLZ5340	*Lipogramma anabantoides*	KX713733	-	-	-	-	genseq-4
USNM 414886	CUR12013	*Lipogramma evides*	KX713750	-	-	-	-	genseq-4
USNM 414889	CUR12031	*Lipogramma evides*	KX713751	KX713834	KX713891	KX713852	KX713872	genseq-4
USNM 414883	CUR12044	*Lipogramma evides*	KX713752	-	-	-	-	genseq-4
USNM 414884	CUR12050	*Lipogramma evides*	KX713753	-	-	-	-	genseq-4
USNM 414887	CUR12078	*Lipogramma evides*	KX713754	-	-	-	-	genseq-4
USNM 414890	CUR12084	*Lipogramma evides*	KX713755	-	-	-	-	genseq-4
USNM 414888	CUR12116	*Lipogramma evides*	KX713757	KX713835	KX713892	KX713853	KX713873	genseq-4
USNM 414882	CUR12118	*Lipogramma evides*	KX713758	-	-	-	-	genseq-4
USNM 414878	CUR12276	*Lipogramma evides*	KX713760	-	-	-	-	genseq-4
USNM 414881	CUR12280	*Lipogramma evides*	KX713761	-	-	-	-	genseq-4
USNM 414885	CUR12281	*Lipogramma evides*	KX713762	KX713837	KX713894	KX713855	KX713875	genseq-4
USNM 414879	CUR12288	*Lipogramma evides*	KX713763	-	-	-	-	genseq-4
USNM 414876	CUR12353	*Lipogramma evides*	KX713767	-	-	-	-	genseq-4
USNM 421602	CUR13100	*Lipogramma evides*	KX713771	-	-	-	-	genseq-4
USNM 426769	CUR13233	*Lipogramma evides*	KX713779	-	-	-	-	genseq-4
USNM 426770	CUR13234	*Lipogramma evides*	KX713780	-	-	-	-	genseq-4
USNM 426771	CUR13265	*Lipogramma evides*	KX713781	-	-	-	-	genseq-4
USNM 426737	CUR13266	*Lipogramma evides*	KX713782	-	-	-	-	genseq-4
USNM 426746	CUR13279	*Lipogramma evides*	KX713785	-	-	-	-	genseq-4
USNM 426709	CUR13286	*Lipogramma evides*	KX713786	-	-	-	-	genseq-4
USNM 426722	CUR13294	*Lipogramma evides*	KX713787	-	-	-	-	genseq-4
Photo Voucher Only	CUR15032	*Lipogramma evides*	KX713793	-	-	-	-	genseq-5
Photo Voucher Only	CUR15055	*Lipogramma evides*	KX713795	-	-	-	-	genseq-5
Photo Voucher Only	CUR15057	*Lipogramma evides*	KX713796	-	-	-	-	genseq-5
Photo Voucher Only	CUR15060	*Lipogramma evides*	KX713798	-	-	-	-	genseq-5
Photo Voucher Only	CUR15061	*Lipogramma evides*	KX713799	-	-	-	-	genseq-5
USNM 434771	CUR15091	*Lipogramma evides*	KX713811	-	-	-	-	genseq-4
USNM 434783	CUR15103	*Lipogramma evides*	KX713813	-	-	-	-	genseq-4
USNM 434784	CUR15104	*Lipogramma evides*	KX713814	-	-	-	-	genseq-4
USNM 431313	TIK003	*Lipogramma evides*	KX713822	-	-	-	-	genseq-4
USNM 422670, paratype	CUR13158	*Lipogramma haberi*	KX713775	-	-	KX713860	-	genseq-2
USNM 422679, holotype	CUR13171	*Lipogramma haberi*	KX713776	-	-	KX713861	-	genseq-1
USNM 434772, paratype	CUR15092	*Lipogramma haberi*	KX713812	-	-	-	-	genseq-2
USNM 406013	CUR11013	*Lipogramma klayi*	KX713737	KX713826	KX713883	KX713845	KX713865	genseq-3
USNM 406133	CUR11133	*Lipogramma klayi*	KX713740	KX713828	KX713885	KX713847	KX713867	genseq-3
USNM 406134	CUR11134	*Lipogramma klayi*	KX713741	-	-	-	-	genseq-3
USNM 406375	CUR11375	*Lipogramma klayi*	KX713744	-	-	-	-	genseq-3
USNM 406376	CUR11376	*Lipogramma klayi*	KX713745	KX713830	KX713887	KX713849	KX713869	genseq-3
USNM 422669	CUR13112	*Lipogramma klayi*	KX713772	-	-	-	-	genseq-3
USNM 422676	CUR13113	*Lipogramma klayi*	KX713773	-	-	-	-	genseq-3
USNM 422690	CUR13114	*Lipogramma klayi*	KX713774	-	-	-	-	genseq-3
Photo Voucher Only	CUR15064	*Lipogramma klayi*	KX713800	-	-	-	-	genseq-5
Photo Voucher Only	CUR15066	*Lipogramma klayi*	KX713801	-	-	-	-	genseq-5
Photo Voucher Only	CUR15068	*Lipogramma klayi*	KX713802	-	-	-	-	genseq-5
Photo Voucher Only	CUR15069	*Lipogramma klayi*	KX713803	-	-	-	-	genseq-5
Photo Voucher Only	CUR15070	*Lipogramma klayi*	KX713804	-	-	-	-	genseq-5
Photo Voucher Only	CUR15075	*Lipogramma klayi*	KX713805	-	-	-	-	genseq-5
Photo Voucher Only	CUR15076	*Lipogramma klayi*	KX713806	-	-	-	-	genseq-5
Photo Voucher Only	CUR15077	*Lipogramma klayi*	KX713807	-	-	-	-	genseq-5
Photo Voucher Only	CUR15084	*Lipogramma klayi*	KX713810	-	-	-	-	genseq-5
USNM 438687	DOM16036	*Lipogramma klayi*	KX713817	-	-	-	-	genseq-4
USNM 438741	DOM16090	*Lipogramma klayi*	KX713819	-	-	-	-	genseq-4
USNM 438742	DOM16091	*Lipogramma klayi*	KX713820	-	-	-	-	genseq-4
USNM 438803	DOM16152	*Lipogramma klayi*	KX713821	-	-	-	-	genseq-4
USNM 406011	CUR11011	*Lipogramma levinsoni*	KX713735	-	-	-	-	genseq-3
USNM 406012	CUR11012	*Lipogramma levinsoni*	KX713736	-	-	-	-	genseq-3
USNM 406018, paratype	CUR11018	*Lipogramma levinsoni*	KX713738	KX713827	KX713884	KX713846	KX713866	genseq-2
USNM 406019	CUR11019	*Lipogramma levinsoni*	KX713739	-	-	-	-	genseq-3
USNM 406139, holotype	CUR11139	*Lipogramma levinsoni*	KX713742	KX713829	KX713886	KX713848	KX713868	genseq-1
USNM 406140, paratype	CUR11140	*Lipogramma levinsoni*	KX713743	-	-	-	-	genseq-2
USNM 406393, paratype	CUR11393	*Lipogramma levinsoni*	KX713747	KX713832	KX713889	KX713851	KX713871	genseq-2
USNM 406394	CUR11394	*Lipogramma levinsoni*	KX713748	-	-	-	-	genseq-3
USNM 426784, paratype	CUR13183	*Lipogramma levinsoni*	KX713777	-	-	-	-	genseq-2
USNM 426754	CUR13184	*Lipogramma levinsoni*	KX713778	-	-	-	-	genseq-3
USNM 426774	CUR13267	*Lipogramma levinsoni*	KX713783	-	-	-	-	genseq-3
USNM 426730	CUR13268	*Lipogramma levinsoni*	KX713784	-	-	-	-	genseq-3
Photo Voucher Only	CUR15031	*Lipogramma levinsoni*	KX713792	-	-	-	-	genseq-5
Photo Voucher Only	CUR15058	*Lipogramma levinsoni*	KX713797	-	-	-	-	genseq-5
USNM 438703	DOM16052	*Lipogramma levinsoni*	KX713818	-	-	-	-	genseq-4
USNM 440439, holotype	CUR16008	*Lipogramma barrettorum*	MG676227	-	-	-	-	genseq-1
USNM 406392, paratype	CUR11392	*Lipogramma barrettorum*	KX713746	KX713831	KX713888	KX713850	KX713870	genseq-2
UF 239254, paratype	CUR11426	*Lipogramma barrettorum*	KX713749	KX713833	KX713890	-	-	genseq-2
USNM 414914, paratype	CUR12149	*Lipogramma barrettorum*	KX713759	KX713836	KX713893	KX713854	KX713874	genseq-2
USNM 431687, paratype	CUR14079	*Lipogramma barrettorum*	KX713789	-	-	-	-	genseq-2
USNM 436460, paratype	CUR15125	*Lipogramma barrettorum*	KX713815	-	-	-	-	genseq-2
USNM 436474, paratype	CUR15139	*Lipogramma barrettorum*	KX713816	-	-	-	-	genseq-2
USNM 431722, holotype	CUR14114	*Lipogramma schrieri*	KX713790	-	-	-	-	genseq-1
USNM 414913, paratype	CUR12101	*Lipogramma schrieri*	KX713756	-	-	-	-	genseq-2
USNM 414911, paratype	CUR12316	*Lipogramma schrieri*	KX713765	KX713839	KX713896	KX713857	KX713877	genseq-2
UF 239255, paratype	CUR12317	*Lipogramma schrieri*	KX713766	KX713840	KX713897	KX713858	KX713878	genseq-2
USNM 430035, paratype	CUR13329	*Lipogramma schrieri*	KX713788	-	-	-	-	genseq-2
USNM 435299, paratype	CUR15012	*Lipogramma schrieri*	KX713791	-	-	-	-	genseq-2
USNM 413864, paratype	CUR12290	*Lipogramma schrieri*	KX713764	KX713838	KX713895	KX713856	KX713876	genseq-2
Photo Voucher Only	BLZ8127	*Lipogramma trilineata*	JQ841643	-	-	-	-	genseq-5
Photo Voucher Only	BLZ8128	*Lipogramma trilineata*	JQ841642	-	-	-	-	genseq-5
USNM 415245	BLZ8168	*Lipogramma trilineata*	JQ841645	-	-	-	-	genseq-4
USNM 415298	BLZ8274	*Lipogramma trilineata*	JQ841646	KX713825	KX713882	KX713844	KX713864	genseq-4
Photo Voucher Only	BLZ8343	*Lipogramma trilineata*	JQ841644	-	-	-	-	genseq-5
USNM 404204	BLZWF204	*Lipogramma trilineata*	KX713734	-	-	-	-	genseq-4
USNM 414989	CUR13082	*Lipogramma trilineata*	KX713768	-	-	-	-	genseq-3
USNM 414990	CUR13089	*Lipogramma trilineata*	KX713769	-	-	-	-	genseq-3
USNM 414991	CUR13090	*Lipogramma trilineata*	KX713770	KX713841	KX713898	KX713859	KX713879	genseq-3
Photo Voucher Only	CUR15034	*Lipogramma trilineata*	KX713794	-	-	-	-	genseq-5
Photo Voucher Only	CUR15078	*Lipogramma trilineata*	KX713808	-	-	-	-	genseq-5
Photo Voucher Only	CUR15079	*Lipogramma trilineata*	KX713809	-	-	-	-	genseq-5
USNM 442762	EUS17109	*Lipogramma regium*	MG676228					genseq-4

## Supplementary materials

Supplementary material 1 Figure S1 Data type: (measurement/occurence/multimedia/etc.) Explanation note: Neighbor-joining network based on COI sequences of *Lipogramma* species investigated in this study. Scale-bar units are substitutions per site. This is an open access article distributed under the terms of the Creative Commons Attribution License (CC BY 4.0), which permits unrestricted use, distribution, and reproduction in any medium, provided the original author and source are credited.Carole C. Baldwin, Luke Tornabene, D. Ross Robertson, Ai Nonaka, R. Grant Gilmore
