# Supplementary figures and images for: More new deep-reef basslets (Teleostei, Grammatidae, Lipogramma), with updates on the eco-evolutionary relationships within the genus
Source: Zookeys. 2018 Jan 16;(729):129–61. doi: 10.3897/zookeys.729.21842 (PMC5811657; doi:10.3897/zookeys.729.21842)

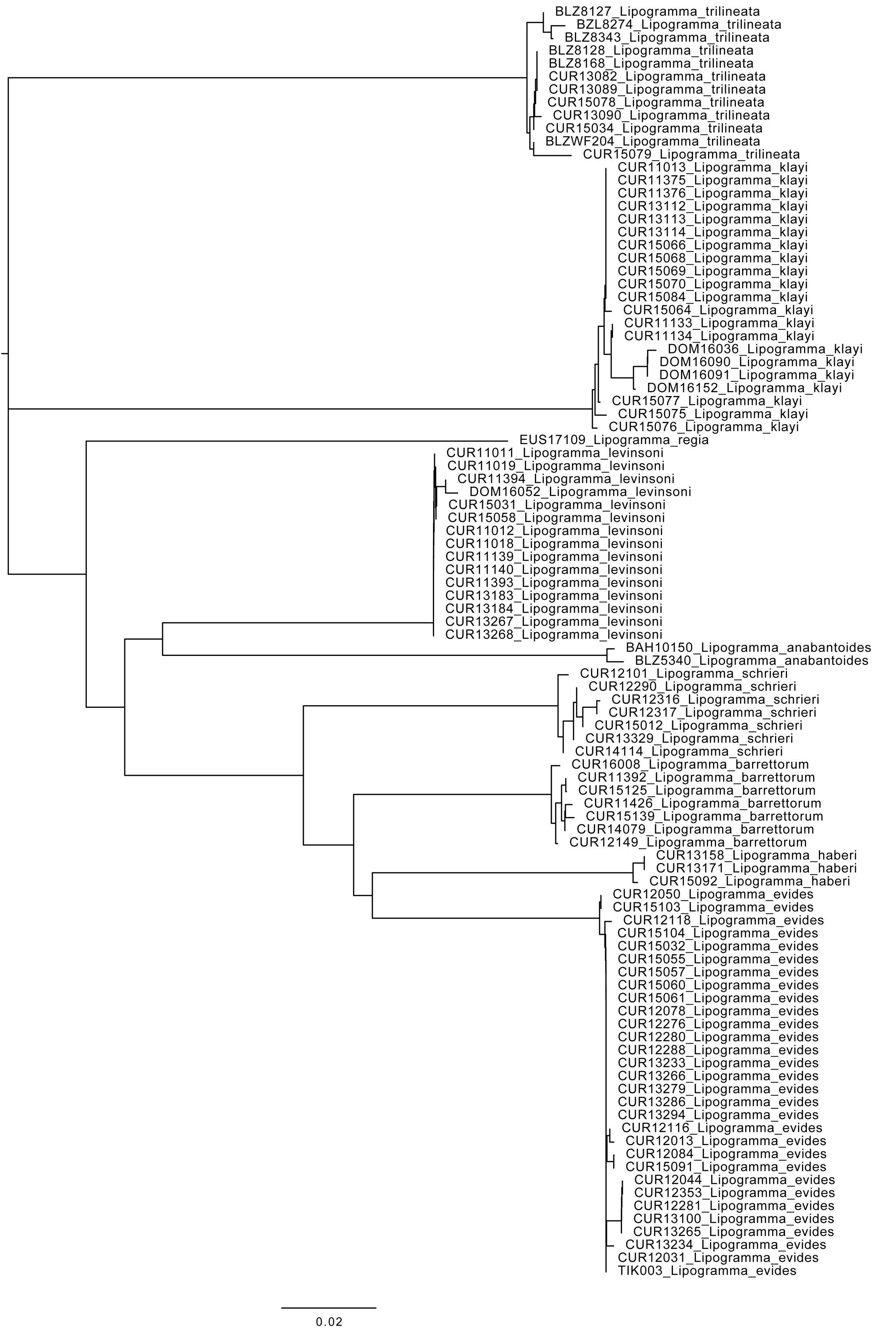

Supplement: Supplementary material 1 — Figure S1 [file zookeys-729-129-s001.tif]
